# Quality control of intraoperative autologous blood salvage in cardiac surgery

**DOI:** 10.1111/trf.18384

**Published:** 2025-08-18

**Authors:** Sergio Domingos Vieira, Fernanda da Cunha Vieira Perini, Leandro Felipe Figueiredo Dalmazzo, Fabiana Akil, Pablo Raphael Gomiero Alves, Erika Abdon Fiquene Oliveira, Marcello Augusto Cesar, Tatiana Covas Pereira, Claudia Monteiro, Mara Cabral Moraes, Katia Beatrice Lima Buratta, Neila Jones Moitinho

**Affiliations:** ^1^ Blood Bank Grupo Gestor de Serviços de Hemoterapia—Grupo GSH São Paulo Brazil

**Keywords:** blood transfusion, autologous, cardiac surgical procedures, operative blood salvage, quality control

## Abstract

**Background:**

The management of intraoperative autologous blood salvage (ABS) quality is essential to ensure safety and outcomes; although publications on this subject are scarce. We evaluated a standardized program of ABS quality control for cardiac and evaluated surgery predictors of mortality in this population.

**Methods:**

Data from a multicenter retrospective study conducted in 27 institutions from January 2021 to December 2023 were analyzed. The quality control of washed autologous red blood cell concentrates that were recovered and reinfused into the patients was assessed directly from the reinfusion bag. Main quality indicators were hematocrit between 50% and 75%; hemolysis level <0.8%; and residual protein <0.5 g/u.

**Results:**

A total of 424 patients and 225,814 mL of intraoperatively recovered washed autologous red blood cells were included, corresponding to 576.6 autologous units. We obtained compliant results in 94.6% for hematocrit, 98.3% for hemolysis levels, and 98.4% for residual protein. A total of 36 (8%) altered results were observed, with most cases (23% or 5.4%) related to hematocrit. Patients requiring homologous blood components were younger (*p* = .006), had lower body weights (congenital heart disease), were female (*p* <.001), had lower preoperative hematocrit and hemoglobin levels (*p* <.001), and experienced more intraoperative or perioperative complications (*p* <.05). The multivariable model yielded significant results for preoperative hemoglobin, hematocrit quality control, intrahospital complications, and blood units transfused.

**Conclusion:**

ABS is essential for the blood conservation program in cardiac surgery. Preoperative anemia, altered quality control, the need for homologous blood transfusion, and the occurrence of clinical and surgical complications impact patient outcomes.

**Abbreviations**
ABS: autologous blood salvage
CPB: cardiopulmonary bypass
ECMO: extracorporeal menbrane oxygenation
LVAD: left ventricular assist devices
TGA: transposition of the great arteries
T4F: Tetralogy of Fallot

## 1 INTRODUCTION

Patients undergoing cardiac surgery, with or without cardiopulmonary bypass (CPB), have a higher risk of excessive bleeding and hemostatic complications due to dilutional effects and the activation and/or consumption of platelets and coagulation factors.^1^ These patients often require a large number of allogeneic blood component transfusions, with transfusion rates varying widely between different centers, ranging from 20% to 67%.^2^

However, the increasing recognition of morbidity and mortality associated with these transfusions in adult^3^, ^4^ and pediatric^5^, ^6^ cardiac surgery patients has heightened interest in and the need for implementing blood conservation programs. Unprocessed autologous blood reinfusion includes unfavorable substances resulting from cytolysis and activation processes, leading to variable and generally poor quality.^7^ Severe and fatal complications have been reported even after transfusion of small amounts of this blood.^8^, ^9^ Recent studies even suggest potential neuropsychological disorders.^10^

Autologous blood salvage (ABS) using an automated cell salvage system is an important and safe component of blood conservation in cardiac surgery with CPB.^11^ Although widely used worldwide, with extensive publications reporting no adverse events^12^, ^13^ or infectious complications,^14^, ^15^, ^16^, ^17^ high quality of the reinfused washed red blood cells is essential. Publications on this topic are scarce, particularly regarding young children, as this procedure was long considered inapplicable to them.^18^ A comprehensive understanding of the procedure, along with appropriate documentation and record‐keeping, is essential to the quality assurance process.^19^, ^20^

We sought to evaluate an ABS quality control system. We also evaluated whether patient outcomes, including mortality, are correlated with the use of homologous blood components during hospitalization and alterations in the quality control of the recovered blood.

## 2 METHODS

This retrospective, cross‐sectional study analyzed data from 27 hospitals across four states in Brazil, evaluating 424 patients who underwent cardiac surgeries, both in adults and in congenital heart disease cases, between January 2021 and December 2023. These patients underwent intraoperative ABS, and their respective quality control of the recovered and reinfused autologous washed red blood cell concentrates was assessed. Data were collected through chart review.

### 2.1 Inclusion and exclusion criteria

All patients undergoing elective cardiac surgery with CPB were evaluated, regardless of underlying risk factors or type of cardiac surgery. Our multicenter cohort included nearly all major types of cardiac surgery performed worldwide, including coronary artery bypass grafting, valve surgery, combined valve and coronary revascularization, aortic aneurysm repair, heart transplantation, Bentall de Bono procedure, cardiac tumor resection, atrial and ventricular septal defect repairs, blood flow redirection procedures, transposition of the great arteries (TGA) repair (Jatene procedure), Tetralogy of Fallot correction (T4F), bidirectional Glenn procedure, Rastelli procedure, and others. Implantation of left ventricular assist devices (LVAD) and extracorporeal membrane oxygenation (ECMO) support was also included.

Exclusion criteria included patients with incomplete medical records (lacking laboratory values, information on recovered blood volume, type of recovery kit used, transfusion data, etc.) and those who did not have intraoperatively recovered blood volume available for reinfusion.

### 2.2 Blood salvage protocol

This study followed a previously standardized protocol for automated intraoperative ABS systems implemented in our institution years ago. All cell salvage machines used in the study were validated and programmed according to this specific protocol for different bowl sizes and respective flow rates at each phase.

Across the 27 hospitals included in the study, 16 semi‐continuous flow automated ABS devices were used. The Sorin Xtra model was utilized in 409 (96.5%) surgeries, while the Didecco Electa was used in 15 (3.5%). Trained operators performed the procedure under medical supervision. The bowl size was selected according to the patient's blood volume to optimize yield and quality: five newborns used a 55 mL bowl, 92 patients used a 125 mL bowl, and 327 patients used a 225 mL bowl.

Blood was aspirated from the surgical field from the time of skin incision until wound closure using a specific dual‐lumen suction catheter designed for blood collection. Vacuum suction pressure was maintained at ≤100 mmHg to minimize hemolysis during aspiration. Anticoagulation of the recovered blood in the cardiotomy reservoir was achieved using 25,000 IU of heparin in 1000 mL of 0.9% saline solution. The anticoagulant flow rate was adjusted according to the rate of surgical bleeding, and the collected blood was filtered and stored in the reservoir.

In addition to aspirated blood from the surgical field, whenever possible, residual blood from the CPB circuit was also directed to the reservoir to maximize blood salvage. The cell salvage system could operate in manual or automatic modes, with the automatic mode being preferred to ensure standardized, pre‐programmed blood processing with minimal operator intervention.

The separation of recovered blood components primarily depends on the density gradient between them. Since red blood cells are heavier than other blood components, they accumulate near the bowl walls during the filling phase, while smaller and lighter particles remain in the center and are expelled. As the red blood cells fill the bowl, they are subsequently washed and resuspended in 0.9% saline solution, achieving an expected hematocrit value of approximately 60%, before being transferred to a sterile reinfusion bag for intraoperative transfusion.^21^, ^22^

During the perioperative period, all patients requiring additional blood replenishment beyond their autologous blood received homologous, leukocyte‐depleted red blood cell concentrates, as determined by the anesthetic or intensive care teams. Coagulation factors and platelets were administered in response to bleeding, coagulopathy, or low platelet counts.

### 2.3 Quality control of recovered blood

Samples were collected directly from the reinfusion bag of the autologous concentrate for quality control analysis. The quality assessment followed Brazilian regulatory standards, which require hemotherapy services to meet a compliance rate of ≥75% for each quality control parameter. Brazil's regulations were adapted from international technical standards.^23^

The required sample size for this study was calculated with a 95% confidence level and 3% precision, yielding a necessary sample of 384 patients. Since our study analyzed 424 patients, the precision increased to 2.85%.

We adopted the same quality control criteria required for homologous washed red blood cell concentrates obtained from blood donation:

- Hematocrit between 50% and 75%, measured by the automated HumanCount 60 analyzer (impedance method).
- Hemolysis rate <0.8% of erythrocyte mass, measured using the BTS 350 Biosystems spectrophotometer.
- Residual protein <0.5 g/unit, assessed using the BTS 350 Biosystems spectrophotometer (Pyrogallol Red‐Molybdate complex method).

Residual heparin levels were not routinely evaluated, as our 2021 study^24^ found values (≤0.1 IU/mL) consistent with the American Association of Blood Banks (AABB) guidelines, which define an acceptable threshold of <0.5 IU/mL.

### 2.4 Statistical analysis

The main outcomes evaluated were mortality. Clinical complications evaluated included surgical site infection, pneumonia, urinary tract infection, sepsis, embolic events, and postoperative arrythmia. For comparisons, we applied the unpaired Student's *t*‐test or Mann–Whitney test, depending on data distribution probabilities. Associations were verified using the chi‐square test or Fisher's exact test. Unadjusted odds ratios (ORs) with 95% confidence intervals were estimated for each characteristic related to mortality using simple logistic regression. A multivariate logistic regression model was developed to identify independent predictors of patient mortality. Visual inspection of plots for collinearity were done between the variables included in the multivariable analysis. Variables with a *p*‐value <.10 in bivariate analyses were included in the model, using a stepwise backward selection method, with an entry and exit criterion of 5% to select variables for the final model.^25^ Statistical analyses were conducted using IBM‐SPSS for Windows version 22.0.

## 3 RESULTS

Regarding age distribution, 67 patients (15.8%) were children (from newborns to 16 years old), while 357 (84.2%) were adults (Table 1). The mean ages were 3.2 years and 58.6 years, respectively, with corresponding mean weights of 15.4 and 78.9 kg. One patient was excluded because he was transferred to another hospital. Preoperative and intraoperative factors (including the quality control of recovered red blood cells) were identified as potential risk factors for increased transfusion requirements. Personalized protocols are shown in Tables 1 and 2.

**TABLE 1:** Population characteristics (*n* = 424).

Age (years)	49.9 ± 23.7
Male	298 (70.3%)
Weight (kg)	68.9 ± 28.1
State, *n* (%)	
São Paulo	371 (87.5%)
Rio de Janeiro	28 (6.6%)
Bahia	12 (2.8%)
Distrito Federal	13 (3.1%)
Hemoglobin (g/dL)	12.5 ± 2.3
Hematocrit (%)	37.9 ± 6.5
Platelets (thousand/mm^3^)	224.3 ± 81.3
Surgeries (*n*)	
Myocardial revascularization	163
Valvular surgery	141
Aortic aneurysm repair	35
Blood flow redirection	16
Atrial septal defect (ASD) repair	15
Ventricular septal defect (VSD) repair	10
Combined procedures	16
Other surgeries	28

*Note*: Data are presented as mean ± standard deviation or frequency (valid %).

**TABLE 2:** Personalized protocol with flows.

Cycle phases	55 mL bowl	125 mL bowl	225 mL bowl
Filling	75 mL/min	125 mL/min	250 mL/min
Washing	75 mL/min	150 mL/min	250 mL/min
Emptying	150 mL/min	250 mL/min	400 mL/min
Concentration	75 mL/min	125 mL/min	300 mL/min
Return	150 mL/min	125 mL/min	300 mL/min
Washing solution volume	500 mL	500 mL	1000 mL

A total of 225,814 mL of intraoperatively recovered washed red blood cell concentrate was obtained, corresponding to 576.6 autologous units, with a mean salvage of 532.6 mL (532.6 ± 376.8 mL), equivalent to 1.37 units (1.4 ± 1) per patient.

Regarding quality control, we achieved compliant results in 94.6% of hematocrit measurements, 98.3% of hemolysis rates, and 98.4% of residual protein levels. A total of 36 cases (8%) showed altered results, with the majority (23 cases, or 5.4%) related to hematocrit, 12 cases (2.84%) to hemolysis rate, and only 1 case (0.6%) to residual protein (Table 3).

**TABLE 3:** Quality control.

Kit (bowl) used, *n* (%)	
55	5 (1.2%)
125	92 (21.7%)
225	327 (77.1%)
Autologous concentrate (mL)	
Mean ± SD	532.6 ± 376.8
Median (p25; p75)	469 (259; 641.5)
Recovered units	
Mean ± SD	1.4 ± 1
Median (p25; p75)	1.2 (0.7; 1.7)
Machine model, *n* (%)	
Xtra	409 (96.5%)
Electa	15 (3.5%)
Hematocrit quality control (%), *n* (%)	
Normal	401 (94.6%)
Altered	23 (5.4%)
Mean ± SD	63.6 ± 10.1
Median (p25; p75)	65.6 (59.8; 69.6)
Hemolysis degree quality control (%), *n* (%)	
Normal	417 (98.3%)
Altered	7 (1.7%)
Mean ± SD	0.4 ± 0.2
Median (p25; p75)	0.4 (0.2; 0.6)
Residual protein quality control, *n* (%)	
Normal	188 (98.4%)
Altered	3 (1.6%)
Mean ± SD	0.3 ± 0.2
Median (p25; p75)	0.3 (0.1; 0.4)

*Note*: Data are presented as mean ± standard deviation (SD), median (25% percentile; 75% percentile), or frequency (valid %), as appropriate.

There was no need for blood transfusion or any blood component in 205 patients (48.3%) in the operating room (OR), 307 patients (72.7%) in the intensive care unit (ICU), and 381 patients (96%) in the general ward. A total of 163 patients (46.3%) did not receive any blood units during the entire hospitalization. The most commonly transfused component was red blood cell concentrate, with a total of 796 units transfused.

Regarding clinical complications, 45 events were reported in the OR, with excessive bleeding being the most frequent. In the ICU, 190 complications were reported, with arrhythmia being the most common. A total of 30 patients (7.1%) died, with 2 deaths occurring in the operating room (Table 4).

**TABLE 4:** Blood product use and clinical outcomes.

Operating room (OR)	
Blood Use in OR, *n* (%)	219 (51.7%)
pRBC (units): (*N* = 165)	2.2 ± 1.3
FFP (units): (*N* = 85)	1.8 ± 0.7
CP (units): (*N* = 117)	6.1 ± 3.7
APC (units): (*N* = 15)	1.1 ± 0.3
Cryo (units): (*N* = 9)	6.9 ± 2.5
Complications in OR, *n* (%)	45 (10.6%)
Intraoperative death, *n* (%)	2 (0.5%)
Intensive care unit (ICU)	
Blood Use in ICU, *n* (%)	115 (27.3%)
pRBC (units): (*N* = 106)	3.9 ± 6.4
FFP(units): (*N* = 26)	3.7 ± 2.5
PC (units): (*N* = 25)	10.9 ± 8.5
APC (units): (*N* = 2)	2 ± 1.4
Cryo (units): (*N* = 11)	4.7 ± 4
Complications in ICU, *n* (%)	113 (26.8%)
Ward	
Blood use in ward, *n* (%)	16 (4%)
pRBC (units) ward (*N* = 13)	2.6 ± 3.7
FFP (units) ward (*N* = 1)	4 ± 0
PC (units) ward (*N* = 4)	7.8 ± 1.9
Discharge type, *n* (%)	
Hospital discharge	373 (88%)
Transferred/ignored	21 (5%)
Death	30 (7.1%)

*Note*: Data are presented as mean ± standard deviation or *n* (valid %).

Abbreviations: APC, autologous platelet; FFP, fresh frozen plasma; PC, platelet concentrate; pRBC, packed red blood cells.

A total of 163 patients (43.6%) did not receive any allogeneic blood transfusion. These patients had preoperative laboratory values with no signs of anemia, with a mean hematocrit of 39.5% and a mean hemoglobin level of 13 g/dL. Among them, 26 patients (15.9%) experienced complications, and 2 patients (1.2%) died. Conversely, the 234 remaining patients (56.4%) who required allogeneic blood transfusion had a mean hematocrit of 36.8% and a mean hemoglobin level of 12.1 g/dL. Among them, 87 patients (37.1%) experienced complications, and 28 patients (11.9%) died.

Regarding the use of allogeneic blood, the data indicate that patients requiring blood components were more likely female (*p* <.001), younger (*p* = .006), had lower body weight (congenital heart disease), had lower preoperative hematocrit and hemoglobin levels (*p* <.001), received higher volumes and units of recovered autologous red blood cell concentrate (*p* = .03 and *p* = .008, respectively), and experienced more complications in the operating room, ICU, and overall (*p* <.05). (Table 5).

**TABLE 5:** Univariate analyses of mortality predictors.

	Death			95% confidence interval		*p‐*value
Variable	No	Yes	OR	Lower	Upper	
Age (years)	50.2 ± 23	46.5 ± 32.5	0.99	0.98	1.01	.70^a^
Sex (male)	278 (93.3%)	20 (6.7%)	0.84	0.38	1.84	.65^b^
Weight (kg)	69.9 ± 27.2	55.7 ± 36.2	0.99	0.97	1	.016^a^
Hematocrit (%)	38.1 ± 6.2	35.1 ± 8.6	0.93	0.88	0.99	.015^c^
Hemoglobin (g/dL)	12.6 ± 2.2	11.6 ± 3	0.83	0.7	0.97	.02^c^
Autologous concentrate (mL)	513.6 ± 319.8	781.9 ± 784.8	1	1	1	.05^a^
Recovered units	1.3 ± 0.8	2 ± 1.9	1.56	1.18	2.07	.05^a^
Altered hematocrit quality control	19 (82.6%)	4 (17.4%)	3.04	0.96	9.58	.07^d^
Any blood use	230 (89.1%)	28 (10.9%)	9.98	2.35	42.49	<.001^b^
Any complication	110 (80.3%)	27 (19.7%)	23.24	6.91	78.15	<.001^b^
Blood components units	1 (0; 6.3)	12.5 (4; 29)	1.1	1.06	1.14	<.001^b^

*Note*: Data are presented as mean ± standard deviation, median (interquartile range) or *n* (valid %), as appropriate.

^a^ Mann–Whitney test.

^b^ Chi‐squared test.

^c^ T‐test.

^d^ Fisher's exact test.

Univariate models studying mortality predictors are shown in Table 5. The multivariable model using backwards stepwise regression (Table 6) yielded significant results for preoperative hemoglobin, hematocrit quality control, intrahospital complications, and blood units transfused. No significant collinearity was observed in the multivariable analyses. A 1 g/dL increase in preoperative hemoglobin levels was associated with an 18% reduction in mortality risk (OR 0.82, 95%CI 0.68–1.0). Patients with altered hematocrit quality control (QC) had a 7.34‐fold higher mortality risk compared to those with normal QC (OR 7.34, 95%CI 1.08–49.95). The occurrence of any complication during hospitalization was strongly associated with mortality (OR = 80.7, 95%CI 7.7–842.9). Each additional unit of transfused blood increased the mortality risk by 8% (OR 1.08, 95%CI 1.05–1.12). (Table 6).

**TABLE 6:** Multivariable regression for mortality predictors.

	OR	95% CI		*p*‐value
Hemoglobin (g/dL)	0.82	0.68	1	.046
Altered hematocrit quality control	7.34	1.08	49.95	.042
Any complication	80.69	7.72	842.88	<.001
Number of blood components (units)	1.08	1.05	1.12	<.001

*Note*: Variables significant on the univariate analyses were included and selected using backwards stepwise selection.

Abbreviations: CI, confidence interval; OR, odds ratio.

## 4 DISCUSSION

This retrospective study analyzed over 400 patients submitted to intraoperative blood salvage and transfusion. It demonstrated that receiving units that fail quality control are one of the main factors associated with the mortality of patients, alongside low preoperative hemoglobin, occurrence of intrahospital complications, and number of units transfused.

Although intraoperative blood salvage using automated cell salvage systems is widely implemented and has been extensively studied in adults^26^, ^27^ and children,^28^, ^29^ and is recommended in guidelines from specialist medical societies,^11^, ^30^ the quality control of the recovered product, particularly its magnitude and impact on patient outcomes, remains understudied.

Anemia is a frequent finding in patients undergoing cardiac surgery. A large‐scale study demonstrated that nearly one‐third of patients had preoperative anemia, and unadjusted mortality and morbidity rates were nearly three times higher in anemic patients. After adjusting for preoperative differences, low preoperative hemoglobin was identified as an independent predictor of mortality and morbidity.^31^ Anemia has been recognized as a significant predictor of adverse outcomes in patients undergoing coronary artery surgery, as well as in mixed cardiac surgical populations.^32^, ^33^ In our study, a low preoperative hemoglobin level was associated with higher mortality.

We evaluated quality indicators for cell salvage, specifically hematocrit, hemolysis rate, and residual protein levels in the recovered blood units. These parameters are mandated by Brazilian regulations for homologous washed red blood cell concentrates, requiring hemotherapy services to maintain a compliance rate of ≥75%, as previously described. Regarding international standards, we suggest a Statistical Sampling for Quality Control be carried out, as recommended by AABB, of at least 1% of the recovered units produced monthly, or at least in 4 procedures per month (whichever is greater).^23^

This study has several limitations. Due to its retrospective nature, the analyses are subject to selection bias. There is also significant heterogeneity of surgical teams; included procedures and indications for transfusion are not uniform across all centers. Our results do not necessarily apply to other scenarios outside of cardiac surgery.^34^ Individualized data regarding other quality markers (protein and hemolysis) were not available for analyses. Further studies with greater methodological rigor, based on well‐established protocols, are necessary. However, the study also has significant strengths. It is a very large sample of real‐world data, demonstrating the actual efficacy and pragmatic outcomes in this population.

## 5 CONCLUSION

Our results demonstrate that autologous blood salvage in cardiac surgery is an effective procedure for reducing the need for allogeneic blood transfusion, playing a crucial role in patient blood management during surgery. Preoperative anemia, altered quality control of the autologous blood unit, the need for blood transfusion, and the occurrence of clinical and surgical complications increase mortality in these patients.

## CONFLICT OF INTEREST STATEMENT

The authors declare no conflicts of interest.
